# Apoptosis regulation by subcellular relocation of caspases

**DOI:** 10.1038/s41598-018-30652-x

**Published:** 2018-08-15

**Authors:** Evgeniia A. Prokhorova, Gelina S. Kopeina, Inna N. Lavrik, Boris Zhivotovsky

**Affiliations:** 1 Faculty of Medicine, Lomonosov Moscow State University, 119991 Moscow, Russia; 2 Translational Inflammation Research, Medical Faculty, Otto von Guericke University, 39106 Magdeburg, Germany; 3 Division of Toxicology, Institute of Environmental Medicine, Karolinska Institutet, Box 210, 17177 Stockholm, Sweden; 4 Present Address: Sir William Dunn School of Pathology, University of Oxford, OX1 3RE Oxford, UK

## Abstract

The cleavage of nuclear proteins by caspases promotes nuclear breakdown and, therefore, plays a key role in apoptosis execution. However, the detailed molecular mechanisms of these events remain unclear. To get more insights into the mechanisms of nuclear events during apoptosis we set up a rapid fractionation protocol for the separation of the cytoplasmic and nuclear fractions of cells undergoing cisplatin-induced apoptosis. Importantly, nuclear accumulation of effector caspase-3 as well as initiator caspase-2, -8 and -9 was observed using the developed protocol and immunofluorescence microscopy. The detection of caspases and their cleavage products in the nucleus occurred within the same time interval after cisplatin treatment and took place shortly before nuclear fragmentation. The entry of initiator caspases to the nucleus was independent of caspase-3. Given that all three initiator caspases had catalytic activity in the nuclei, our findings indicate that initiator caspases might participate in the proteolysis of nuclear components during apoptosis, promoting its disintegration and apoptotic cell death.

## Introduction

Apoptosis is the best known mode of programmed cell death that is crucial for tissue development and homeostasis^1^. One of the hallmarks of apoptosis is the breakdown of the cell nucleus^2^. This multi-step process comprises chromatin condensation, DNA fragmentation and nuclear envelope collapse. Caspases, a family of cysteine-dependent proteases, play important role in these events both through the proteolysis-mediated activation of other apoptotic proteins and directly by the cleavage of nuclear proteins^3–6^. Given that the nucleus is the source of potentially immunogenic proteins and nucleic acids, the latter of which could have viral origin or oncogenic properties, its fast demolition and removal represent extremely important steps in the course of apoptotic cells death^7–9^. The main role in the breakdown of the nucleus and other cellular compartments during apoptosis belongs to effector caspase-3, while the role of other caspases in the process remains unclear^10,11^. Importantly, while caspase substrates were reported to be evenly distributed throughout the cell^12^, few nuclear substrates of initiator caspases have been recognized^13–15^.

Caspase-3, as well as other caspases with the exception of caspase-2, do not possess a nuclear localization signal (NLS) required for protein nuclear import by importins (karyopherins)^16^. Accordingly, a number of different mechanisms have been suggested to underlie the nuclear accumulation of caspase-3 during apoptosis, including passive diffusion^17^ and active transport^18–20^. As mentioned above, only caspase-2 was shown to harbor a NLS among all caspases^21–24^. At the same time, although some reports suggested nuclear localization of caspase-8 and -9^13,14,25^, caspase-2 is the only initiator caspase for which nuclear localization has been described as a way of regulating its functions^26,27^. However, the data on caspase-2 cellular distribution and its activation site remain controversial^28–32^, although the recent findings have significantly improved our current understanding^27,33^. In most of the previous studies on caspase-2 localization, the authors used ectopical expression of caspase-2 linked to a fluorescent protein, e.g. GFP, which is reported to translocate to the nucleus on its own^34^. Additionally, often the assessment of caspase-2 localization was conducted only at the late stages of apoptosis, i.e. after 24 hr of treatment with an apoptosis-inducing agent without regard to time-dependent alterations in the nuclear structure during apoptosis^3,6^.

In the present study, we aimed to assess cellular compartmentalization of caspases in the course of DNA damage-induced apoptosis taking into account time-dependent alterations in the nuclear structure during this type of cell death. For this, we set up a rapid subcellular fractionation method allowing efficient separation of cytoplasmic and nuclear components. Using this method, as well as immunofluorescence microscopy, we assessed the changes in the cellular distribution of initiator caspase-2, -8, -9 and effector caspase-3 during apoptosis.

## Results

### Lysis with NP-40 was selected for the separation of cytoplasmic and nuclear components

To investigate the changes in caspase localization during apoptosis considering time-dependent alterations in the nuclear structures, we compared several fractionation approaches and set up a protocol for the fractionation of HeLa and Caov-4 cells. The purity of the fractions was assessed in parallel by two approaches: Western blotting (WB) and DIC/fluorescence microscopy using the staining with Hoechst33342 and ER-tracker Green. For WB analysis, lamin B and PARP1 (poly(ADP-ribose) polymerase 1) were used as nuclear markers, GAPDH (glyceraldehyde 3-phosphate dehydrogenase) and vinculin as cytosolic markers, Na^+^/K^+^ ATPase as a plasma membrane marker, ERp29 (Endoplasmic reticulum protein 29) as an ER marker, and cyt *c* (cytochrome *c*) as a mitochondrial marker.

**Figure 1:**
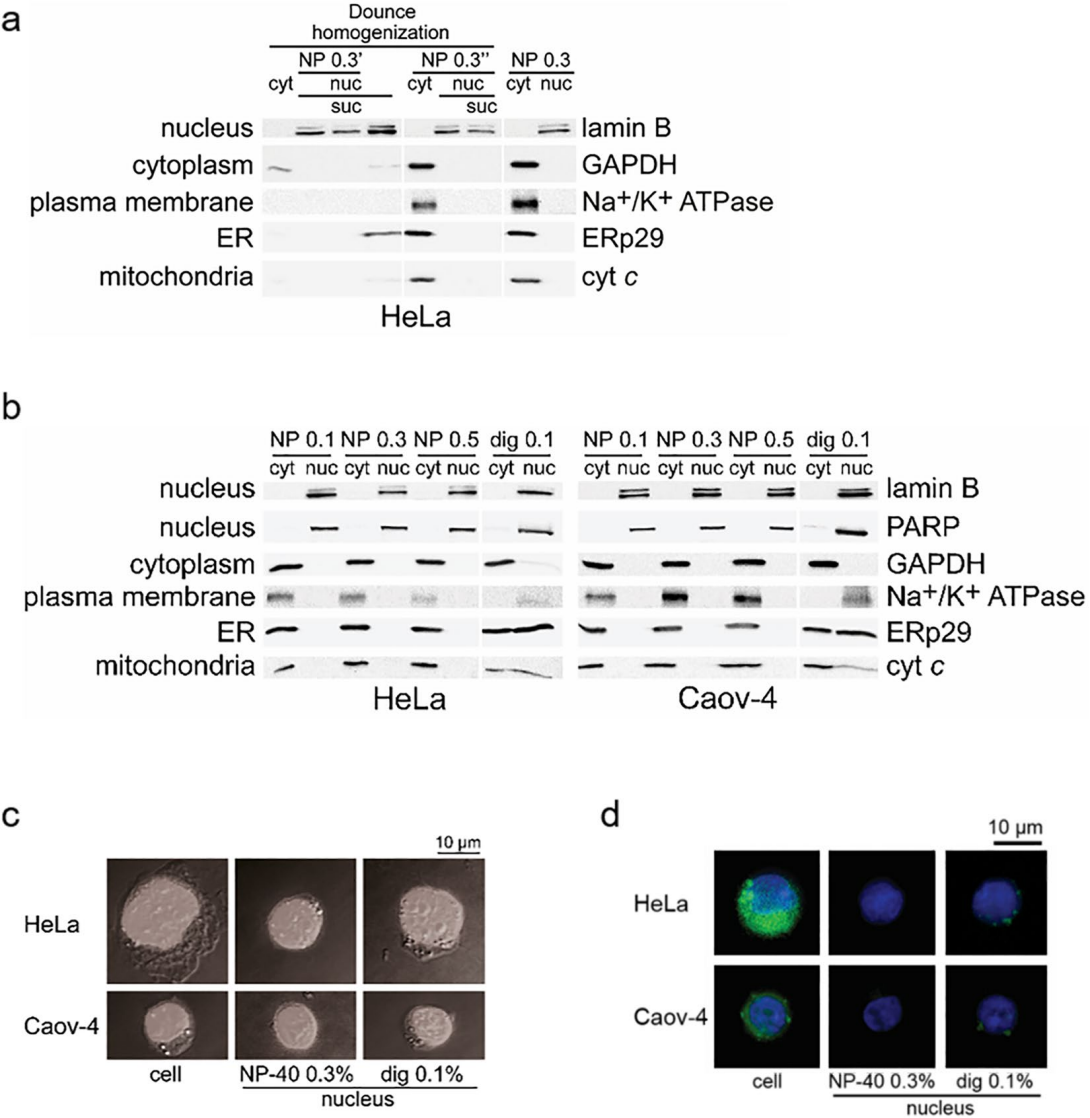
Subcellular fractionation of HeLa and Caov-4 cells. (**a**) Comparison of the cytoplasmic and nuclear fractions of HeLa cells prepared by lysis with NP-40 and/or using Dounce homogeniser and/or centrifugation through a sucrose solution. NP 0.3′, lysis with 0.3% NP-40 after Dounce homogenization; NP 0.3″, simultaneous application of Dounce homogeniser and 0.3% NP-40; NP 0.3, two-step lysis with 0.1% and 0.3% NP-40; suc, sucrose purification. (**b**) WB analysis of the obtained by lysis with a non-ionic detergent cytoplasmic and nuclear fractions. NP, lysis with NP-40, first, at 0.1% and, then, using the indicated percentage for the additional purification of the nuclei; dig, two-step lysis with 0.1% digitonin. (**a**,**b**) Cyt, cytoplasmic extract; nuc, isolated nuclei. Lamin B and PARP1 (nuclear proteins), GAPDH (a cytoplasmic protein), Na^+^/K^+^ ATPase (a plasma membrane protein), ERp29 (an ER protein) and cyt *c* (a mitochondrial protein) served as fractionation markers. (**c**) Cells and isolated nuclei stained with Hoechst33342 and imaged by dual DIC and fluorescence microscopy. (**d**) Cells and isolated nuclei stained with Hoechst33342 and ER-tracker Green (BODIPY FL Glibenclamide) and imaged by fluorescence microscopy. (**c**,**d**) Scale bars, 10 μm.

Dounce homogenization alone showed low efficiency of the extraction of cytoplasmic components (Fig. 1a, lane 1) and did not allow to isolate pure nuclear fraction. In particular, cytosolic and ER markers - GAPDH, ERp29 and cyt *c* were detected in this fraction using WB analysis (Fig. 1a, lane 4). This precluded further application of this approach. In contrast to Dounce homogenization alone, the application of non-ionic detergent NP-40 in the concentration range from 0.1 to 0.5% with and without Dounce homogenization allowed to obtain pure cytoplasmic and nuclear fractions (Fig. 1a and b). Lysis of cells with another non-ionic detergent, digitonin, which was also previously used for the isolation of nuclei^35,36^, allowed to separate the nuclei from the cytosol, but not from the plasma membrane, ER or mitochondrial components (Fig. 1b). The addition of sucrose centrifugation step did not increase fraction purity but lowered the yield of nuclear components (Fig. 1a, lanes 3, 7). Thus, the lysis with NP-40 alone provided the most rapid and efficient separation of pure cytoplasmic and nuclear components. To ensure the purity of the isolated nuclei using 0.3% NP-40, the imaging amalysis with Hoechst33342 and ER-tracker Green has been undertaken. The subsequent analysis with dual DIC and fluorescence microscopy showed the absence of ER-tracker Green-stained components in the nuclei isolated using NP-40, but not in the case of using digitonin (Fig. 1c and d).

Consequently, the fractionation method based on cell lysis with NP-40 was chosen for further preparation of the cytoplasmic and nuclear fractions. Lysis was performed in two steps. First, NP-40 at 0.1% concentration in hypotonic conditions (for increasing the efficiency of plasma membrane collapse) was used for the separation of the cytoplasmic components. Then, the nuclei were purified with 0.3% NP-40 in isotonic conditions (corresponds to Fig. 1b, NP 0.3). This nuclei purification step was introduced as some components, namely ER components, are tightly connected to the outer nuclear membrane and are particularly difficult to detach^37^. Moreover, in accordance with fluorescence microscopy analysis (data not shown) using 0.3% NP-40 allowed to isolate more pure nuclei in comparison with 0.1% NP-40. The higher concentration of NP-40 (0.5%) did not improve the separation of pure cytoplasmic and nuclear components (Fig. 1b, NP 0.3 vs NP 0.5). The major steps of the protocol are depicted in the Fig. S1.

### The time course of cisplatin-induced apoptotic events

To analyze the redistribution of caspases between the cytoplasm and the nucleus during apoptosis, HeLa and Caov-4 cells were treated with cisplatin, a genotoxic drug commonly used against various types of cancer, including cervical and ovarian cancers^38^. Regardless of the nature of the apoptotic stimulus, apoptosis is morphologically characterised by the condensation (hypercondensation of chromatin, or pyknosis) and fragmentation of the nuclei (karyorrhexis)^5^. To distinguish between the stages of the changes in the nuclear morphology, we defined the time course of these alterations in HeLa and Caov-4 cells in response to treatment with 35 μM cisplatin.

**Figure 2:**
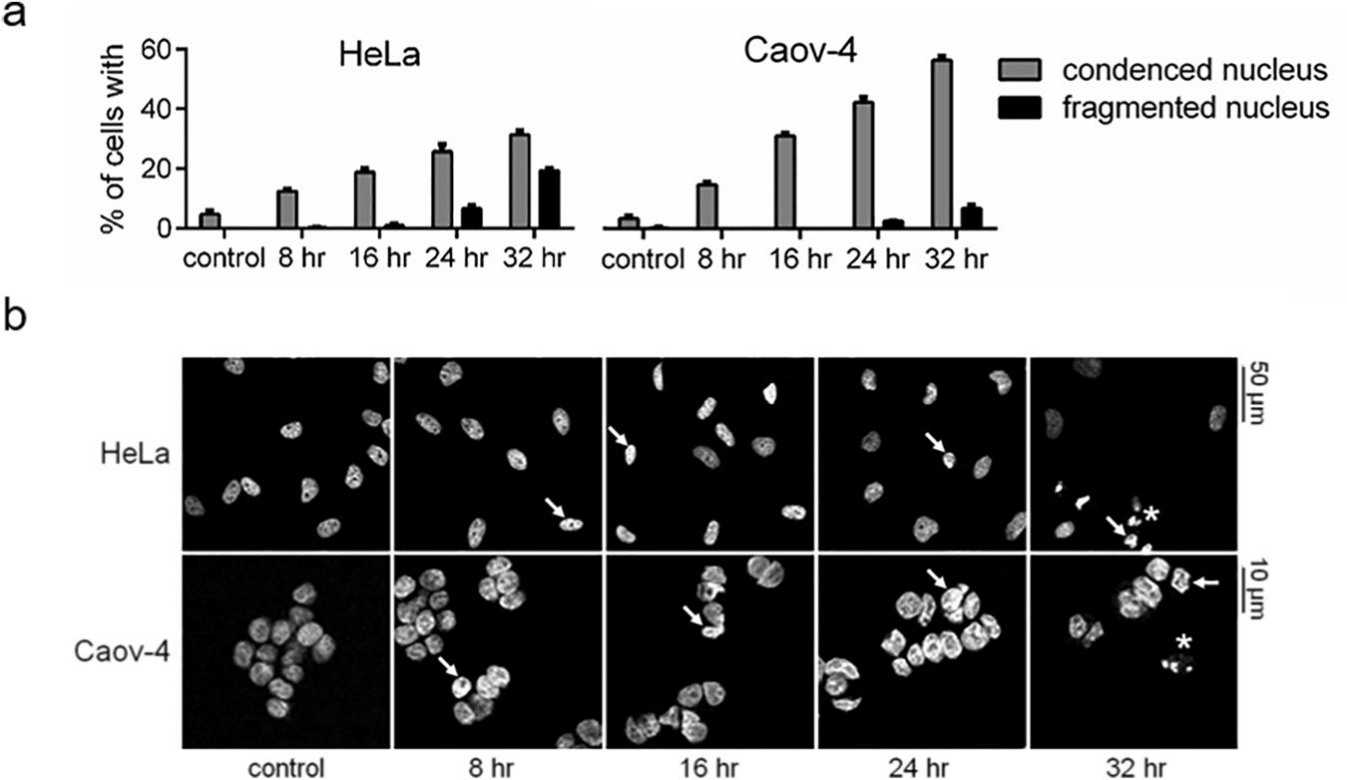
Cisplatin (35 μM)-induced changes in the morphology of HeLa and Caov-4 cells. (**a**) Percentages of cells with condensed and fragmented nuclei detected by DAPI staining. Results are shown as mean ± s.e.m. of three independent experiments. At least 200 stained cells were counted in each experiment. (**b**) Representative images obtained using confocal microscopy. The white arrows and asterisks mark examples of condensed and fragmented nuclei, respectively. Scale bars, 50 and 10 μm.

Staining with DAPI showed a time-dependent increase in the percentage of cells with condensed nuclei, which was monitored by brighter and more compact nuclei than the ones from viable cells (Fig. 2). Whereas after 16 hr of cisplatin treatment nuclear condensation was observed in 18.9 ± 1.2% and 30.4 ± 0.8% of HeLa and Caov-4 cells, respectively, significant numbers of fragmented nuclei (more than 6.0%) were detected only following 24 and 32 hr after cisplatin treatment of HeLa and Caov-4 cells, respectively (Fig. 2a).

### Caspase-2, -3, -8, and -9 accumulate in the nucleus during apoptosis

**Figure 3:**
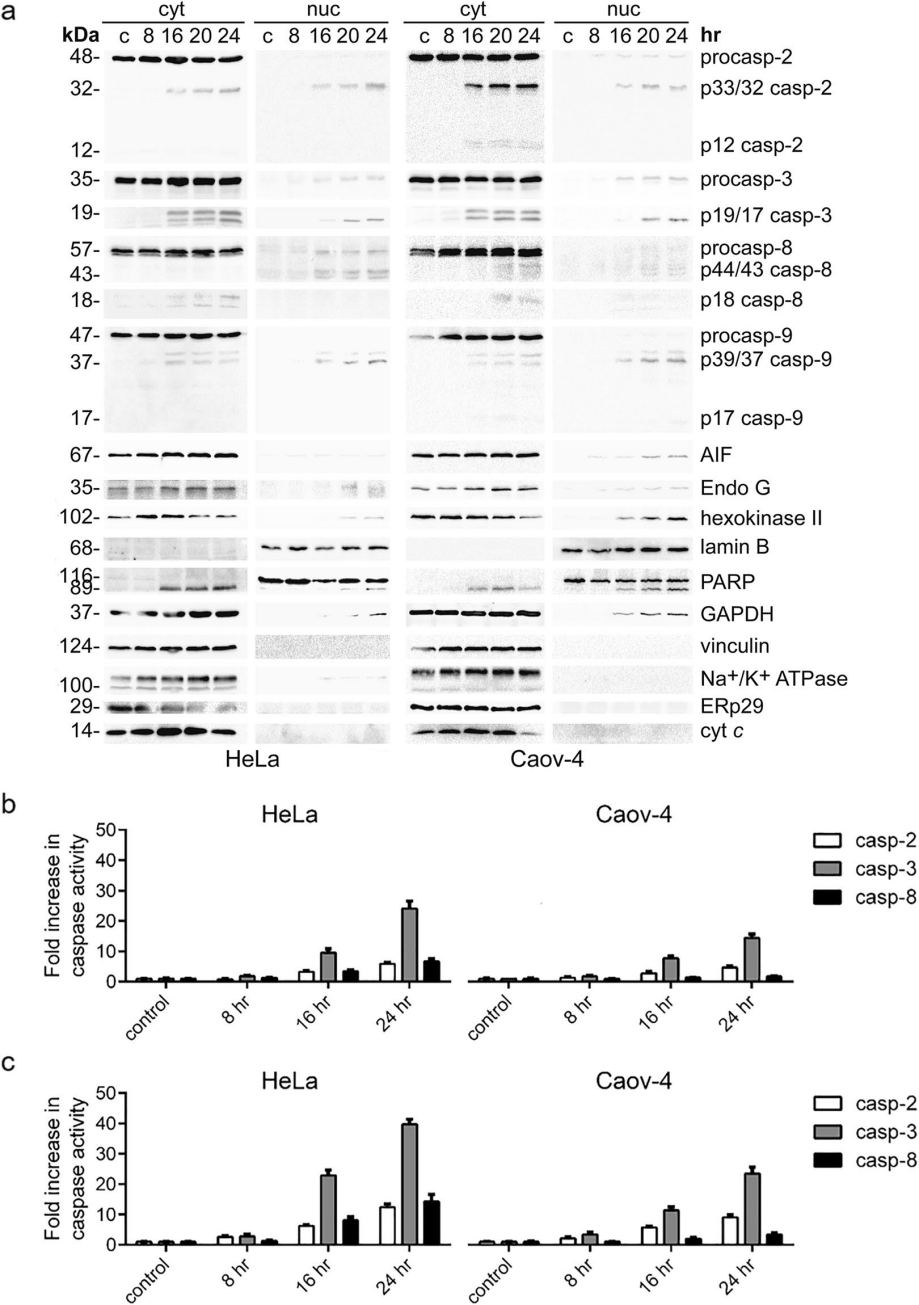
Functionally active initiator caspase-2, -8, -9 and executioner caspase-3 accumulate in the nucleus in response to the treatment of HeLa and Caov-4 cells with cisplatin (35 μM). (**a**) WB analysis of caspase cellular localization upon cisplatin treatment. Representative images of three independent experiments are shown. (**b**,**c**) Caspase activity measurement in the nuclear (**b**) and cellular (**c**) lysates at the indicated time points. Caspase activity was measured by the release of the fluorogenic group AMC from VDVAD-AMC, (fold increase in caspase-2 activity), DEVD-AMC, (fold increase in caspase-3-like activity) and by the release of AFC from IETD-AFC, (fold increase in caspase-8 activity). Results represent the means ± s.e.m. of at least three independent experiments.

The time course of caspase redistribution during cisplatin-induced apoptosis was assessed by WB of their pro- and cleaved forms in the cytoplasmic and nuclear fractions of HeLa and Caov-4 cells. In unstimulated cells, initiator caspase-2, -8, -9 and effector caspase-3 were detected in the cytoplasm (Fig. 3a). After 16 hr treatment with cisplatin, the accumulation of effector caspase-3 and initiator caspase-2 in the nuclear fractions of both cell lines was observed (Fig. 3a). Notably, both caspase-3 and -2 were detected in the nuclear fractions predominantly in their cleaved forms indicating that they might be present in the nuclei of apoptotic cells in the catalytically active state. Furthermore, the cleavage of PARP1 to its 89-kDa fragment in the nucleus has been observed, further supporting the presence of active caspase-3 in this cell compartment.

The measurement of initiator caspase-2 and effector caspase-3 activities with its fluorogenic peptide substrates in the nuclei isolated using the developed fractionation protocol also indicated catalytic activity of these caspases. In particular, an increase of caspase-3 and -2 activities in the nuclear fractions after 16 hr of incubation with cisplatin was detected (Fig. 3b), that is consistent with the results obtained by WB analysis (Fig. 3a, nuc). The timings of caspase activation in the nucleus (Fig. 3a, nuc, Fig. 3b) were similar to those in the cytoplasm (Fig. 3a, cyt) and in total cellular lysates (Fig. 3c).

Interestingly, initiator caspase-8 and -9 were also detected in the nuclear fractions of HeLa and Caov-4 cells after 16 hr treatment with cisplatin (Fig. 3a). The measurement of caspase-8 activity using its fluorogenic peptide substrate also demonstrated an increase in its catalytic activity in the nuclear and cellular lysates following 16 hr incubation with cisplatin (Fig. 3b and c). Thus, effector caspase-3 and all three initiator caspases analyzed showed similar timing of appearance in the nucleus in the course of cisplatin-induced apoptosis.

In addition to the nuclear accumulation of caspases, after 16 hr incubation with cisplatin, we observed the nuclear translocation of several other proapoptotic proteins, namely AIF (Apoptosis-inducing factor), Endonuclease G as well as GAPDH and hexokinase II (Fig. 3a, nuc), and the cytoplasmic entry of the 89-kDa fragment of PARP1 (Fig. 3a, cyt). These translocation events have already been reported in response to a range of apoptotic, as wells as other, stress stimuli^27,39–50^.

At the same time, vinculin, Na^+^/K^+^ ATPase, ERp29, and cyt *c* could not be detected in the nuclear fractions of cisplatin-treated cells (Fig. 3a, nuc). Moreover, no significant decrease in the full-length lamin В levels was observed up to 24 hr of cisplatin treatment (Figs 3a and S3), also indicating that caspases are detected in the nucleus before massive degradation of the nuclear lamina. These data are consistent with the results of DAPI staining and microscopy analysis, which demonstrated only minor levels of nuclear fragmentation at this time point (Fig. 2).

**Figure 4:**
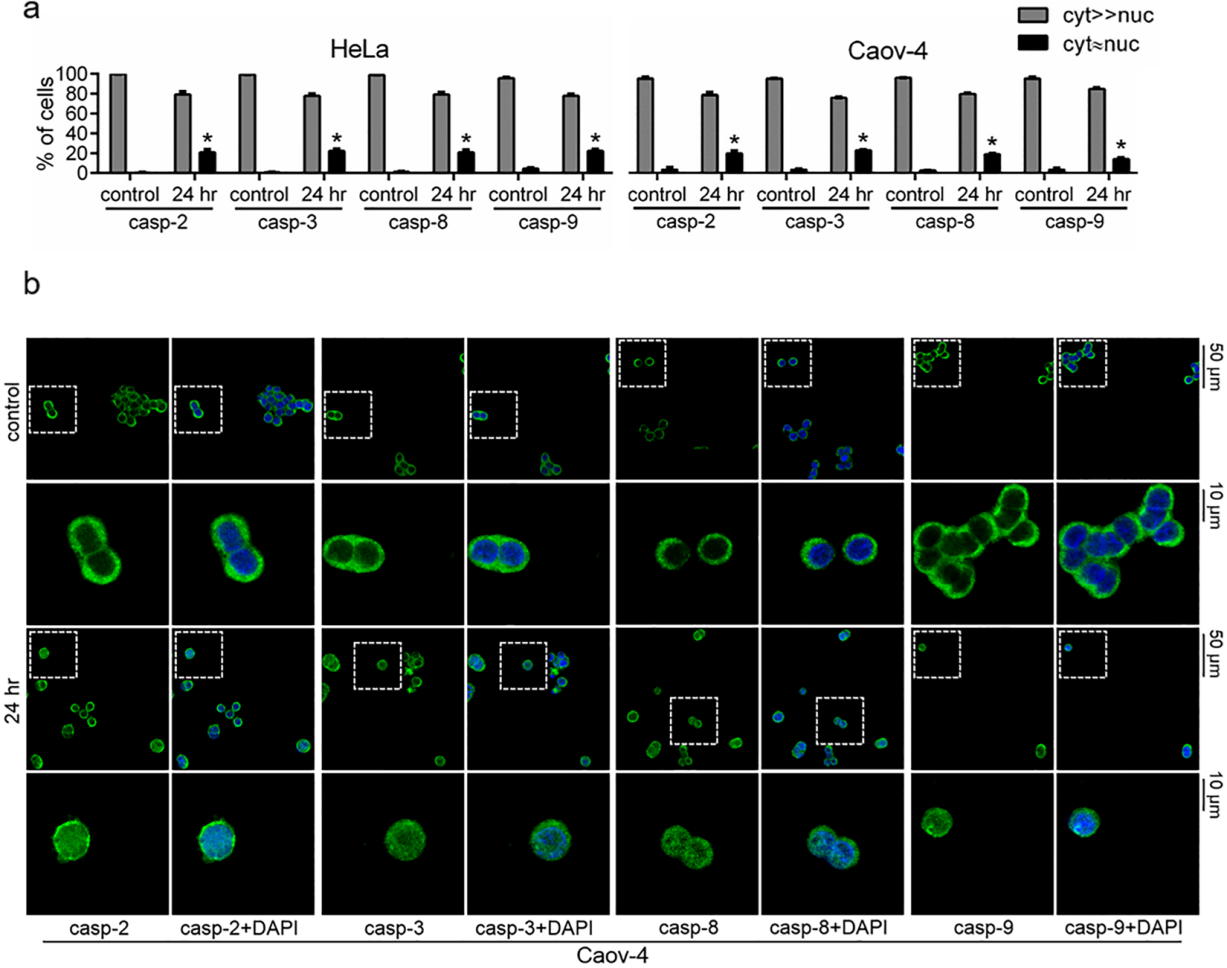
Confocal microscopy analysis of the cellular redistribution of caspase-2, -3, -8 and -9 in response to treatment with cisplatin (35 μM, 24 hr). (**a**) Statistical analysis of nuclear translocation of caspases. Results represent the means ± s.e.m. of three independent experiments. At least 200 cells were counted in each experiment. *p < 0.005. (**b**) Representative images of Caov-4 cells stained with primary anti-caspase-2, -3, -8 or -9 and secondary Alexa Fluor 488 (depicted in green) antibodies. Nuclei were stained with DAPI (shown in blue). Scale bars, 50 and 10 μm.

In order to validate the fractionation results by an independent approach we performed immunofluorescence microscopy analysis. As shown in the Fig. 4a, the nuclear accumulation of caspase-2, -3, -8 and -9 was observed in 20.8 ± 2.7%, 22.2 ± 1.9%, 20.9 ± 2.1% and 22.1 ± 1.6% of treated with cisplatin for 24 hr HeLa cells, respectively (the representative images are shown in the Fig. S4). The similar redistribution patterns were seen for Caov-4 cells −20.0 ± 2.4%, 23.0 ± 0.5%, 19.1 ± 0.8% and 14.2 ± 1.3% for caspase-2, -3, -8 and -9, respectively (Fig. 4). The data are in accordance with the results of the subcellular fractionation, and further confirm that the initiator caspase-2, -8, -9 and effector caspase-3 are present in the nuclei of cisplatin-treated HeLa and Caov-4 cells.

In addition to the assessement of cisplatin-induced changes in the cellular distribution of caspases, we decided to test the effects of two different apoptosis inducers with extranuclear attack points: combined proinflammatory cytokine tumor necrosis factor α (TNFα, 10 ng/ml) and a protein synthesis inhibitor cycloheximide (CHX, 5 μg/ml) treatment, and a broad-specificity protein kinase inhibitor staurosporine (0.1 μM). Both apoptotic stimuli induced the nuclear translocation of initiator caspase-2, -8, -9 and executioner caspase-3 after 4 hr of treatment (Fig. S5a, nuc and S5b, nuc, respectively). As TNFα and staurosporine do not induce direct DNA damage^51^, these results imply that the nuclear translocation of initiator caspases might take place not only during the DNA damage-induced apoptosis, but in response to apoptotic stimuli of diverse mechanisms of action.

Collectively, our findings demonstrate that in the course of apoptotic cell death induced by cisplatin, TNFα/CHX and staurosporine, the initiator caspase-2, -8, -9 and effector caspase-3 translocate to the nucleus, and their translocation precedes the fragmentation of this intracellular compartment.

### The nuclear translocation of initiator caspase-2, -8 and -9 during apoptosis is not dependent on caspase-3

The majority of proteins which are cleaved in the final stages of apoptosis have been reported to be the substrates of caspase-3^11^. At the same time, several works suggested that the nuclear translocation of active caspase-3 might be necessary for the execution of apoptosis, whereas defects in its nuclear entry could underlie potential mechanisms of apoptosis resistance^52–54^. To address whether the appearance of caspases-8, -9 and -2 in the nucleus is also dependent on caspase-3, we analyzed the distribution of initiator caspase-2, -8 and -9 in caspase-3-deficient MCF-7 cells^55^ treated with cisplatin.

**Figure 5:**
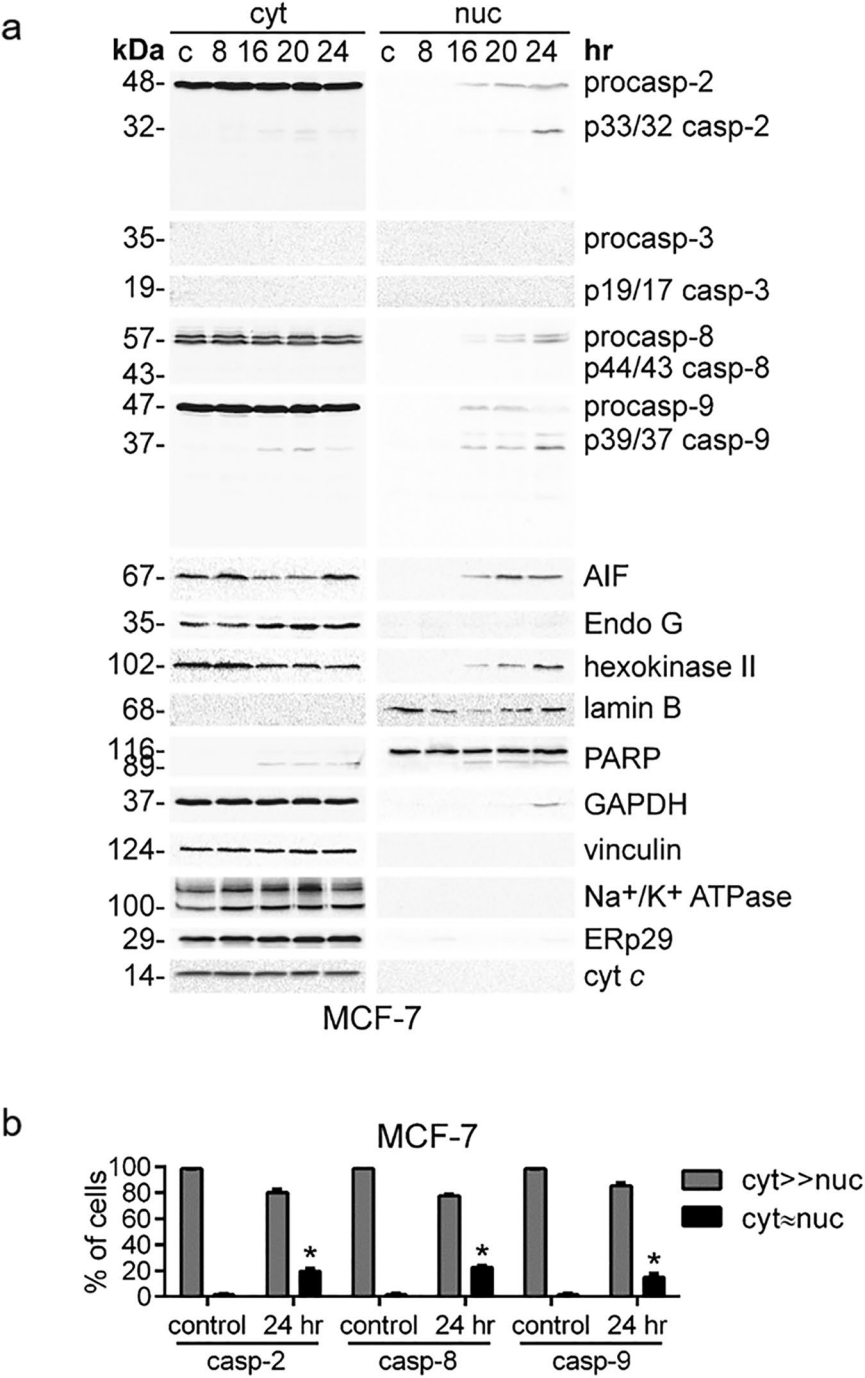
Caspase-3 deficiency in MCF-7 cells does not prevent the translocation of initiator caspases to the nucleus induced by cisplatin (35 μM). (**a**) WB analysis of caspase cellular localization. Representative images of 3 independent experiments are shown. (**b**) Confocal microscopy analysis of the cellular redistribution of initiator caspase-2, -8 and -9 in response to treatment with cisplatin (35 μM, 24 hr). Results represent the means ± s.e.m. of three independent experiments. At least 200 cells were counted in each experiment. *p < 0.005.

The fractionation of MCF-7 cells followed by WB analysis showed that initiator caspase-2, -8 and -9 were detected in the nuclear fraction after 16 hr of cisplatin treatment (Fig. 5a, nuc). Therefore, in caspase-3-deficient MCF-7 cells, the initiator caspases followed the same redistribution pattern in response to cisplatin treatment as in HeLa and Caov-4 cells (Fig. 3a, nuc). Interestingly, no significant differences were observed in the nuclear accumulation of caspase-2, -8 and -9 in the course of cisplatin-induced apoptosis. The purity of the obtained fractions was validated by WB (Fig. 5a) and microscopy analysis (Fig. S6a and b).

The application of immunofluorescence microscopy confirmed these results, clearly showing the accumulation of caspase-2, -8 and -9 in the morphologically integral nuclei of cisplatin-treated cells despite the absence of caspase-3 (Figs 5b and S6c). The numbers of MCF-7 cells showing the nuclear accumulation of caspase-2, -8 and -9 (Fig. 5b) were similar to those observed in HeLa and Caov-4 cells (Fig. 4a). Moreover, caspase-3 deficiency did not affect cisplatin-induced nuclear entry of other proapoptotic proteins analyzed in this study, namely AIF, EndoG, GAPDH and hexokinase II. Thus, the timing of their accumulation in the nuclei of MCF-7 cells (Fig. 5a, nuc) was similar to those observed for HeLa and Caov-4 cells (Fig. 3a, nuc).

In summary, effector caspase-3 and initiator caspase-2, -8 and -9 accumulate into the nucleus during apoptosis with similar accumulation timings. The translocation of initiator caspases is still observed in caspase-3-deficient cells treated with cisplatin, and therefore, does not depend on caspase-3 activity.

## Discussion

The degradation of the nucleus is an extremely important step in apoptotic process^2,5^. Taking into account the key role of caspases in the nuclear demolition, their translocation to the nucleus plays a critical role in the breakdown of this cell compartment and the progression of apoptosis. However, the patterns of caspase redistribution in response to different apoptotic stimuli and molecular mechanisms of these events remain contradictory.

To analyse the changes in caspase localization during apoptosis we set up the fractionation protocol allowing rapid and efficient separation of the cytoplasmic and nuclear components of HeLa, Caov-4, MCF-7, and probably other mammalian cells. Using the developed approach, the nuclear translocation of not only initiator caspase-2 with a NLS, but also of caspase-8 and -9, was demonstrated alongside with the effector caspase-3 in response to the treatment with cisplatin (as well as TNFα/CHX and staurosporine by the fractionation of HeLa cells). Previously, among the initiator caspases, nuclear localization has been reported to play an important role only for caspase-2^26,27^. However, we did not observe any distinct patterns of localization for this caspase in our experiments. Using independent approaches, we have shown that, during apoptosis, caspase-2 enters the nucleus simultaneously with other initiator caspases and caspase-3.

The fact that the patterns of caspase redistribution were similar in all the three cell lines suggests the widespread nature of the observed phenomenon, even though frequently reported cell type-dependent variabilities cannot be excluded and not all of the initiator caspases might translocate into the nucleus in certain cell lines. For instance, caspase-2, -3 and -8, but not -9, were previously detected in the nuclear fraction of Jurkat cells following etoposide and anti-CD95/Fas/Apo-1 antibody treatment^56^, although the study assessed only one time point and used a different antibody against caspase-9. In addition, it was reported that procaspase-8 can be present in the nucleus without apoptotic stimuli and might have non-apoptotic functions, in particular, in DNA repair^57^. Uncovering the crosstalk between apoptotic and non-apoptotic functions of caspases in the nucleus is the question for the future studies. Likewise, the possibility of cell type-dependent variations in caspase nuclear translocation and their role is highly conceivable, and needs to be addressed in the future work.

Of note, all the four caspases were present in the nuclei of apoptotic cells predominantly in their catalytically active state. Possibly, this is because procaspases are less mobile than their cleaved forms. Indeed, the cleavage of procaspases results in the loss of the prodomain and the linker, and leads to the rearrangement of their tertiary structure^58,59^. Other factors, such as the changes in the patterns of caspase post-translational modifications that are observed during apoptosis^60^, could also contribute to the increase in the mobility of cleaved caspases by altering its charge and conformation.

Generally, caspase-3 is considered as the main effector protease that cleaves a large number of substrates, dismantling the nucleus and other cellular compartments in the course of apoptosis^11,61^. However, here we observed similar timings and rates of the nuclear accumulation for both initiator and effector caspases. While the obtained dataset does not contradict the concept that caspase-3 is the major apoptotic protease, it suggests that the role of initiator caspases in the degradation of the nucleus during apoptosis could be underestimated. Thus, it is possible that initiator caspases can cleave a certain range of nuclear proteins, some of which might not even be targeted by effector caspases. In concordance, each of caspase-2, -3, and -7 has been shown to cleave a particular set of substrates at different rates that vary by over 500-fold^12,62^. Furthermore, after their translocation into the nucleus, initiator caspases might promote nuclear proteolysis via the activation of effector caspases directly in this cellular compartment. Initiator caspases might also have functions which are independent of their proteolytic activity, for example, a cleaved product of caspase-8 was shown to translocate to the nucleus and cause the upregulation of genes encoding proapoptotic factors^63^.

Interestingly, cisplatin-induced nuclear entry of initiator caspases did not depend on effector caspase-3, since initiator caspase redistribution patterns remained the same in caspase-3-deficient MCF-7 cells. Although caspase-7 can partially compensate the absence of caspase-3 in some cell types, it is generally accepted that all effector caspases have distinct, non-redundant functions, and the execution of apoptosis in MCF-7 cells is not an exception^11,64^. Due to the crucial role of caspase-3 in apoptotic cell death^11^, the nuclear translocation of initiator caspases seems to be independent not only of the function of caspase-3, but also of other effector caspases. In cells with caspase-3 deficiency or downregulation, the nuclear translocation and function of initiator caspases might be particularly important for the successful execution of apoptosis.

In response to cisplatin, TNFα/CHX and staurosporine, in addition to caspase entry into the nucleus, we detected the cytoplasmic translocation of the apoptotic 89-kDa fragment of PARP1, which is plausible since caspase-mediated cleavage of PARP1 removes its DNA binding domain and disrupts its NLS. Previously, this translocation event has already been described in response to different apoptotic stimuli, including TNFα and DNA-damaging agent etoposide^45–50^. Moreover, we observed the nuclear accumulation of GAPDH and hexokinase II during cisplatin-induced apoptosis. The nuclear entry of GAPDH has also been demonstrated in response to DNA damage^27^, oxidative stress^39,42^ and treatment with TNFα^65^. Although hexokinase II nuclear entry has not yet been demonstrated in mammalian cells, it was reported in yeasts^40,41^. At the same time, cisplatin treatment led to significant accumulation of AIF and Endonuclease G in the nucleus. The movement of these proteins to the nucleus has already been demonstrated in response to a range of apoptotic stimuli and promotes chromatin condensation and DNA fragmentation via caspase-independent apoptotic pathway^43^.

Given that the nuclear membrane permeability is known to increase in the course of apoptotic cell death^3,6^, caspases and other proapoptotic proteins might enter the nucleus to induce its apoptotic demolition independently of specific transport systems. On the other hand, whereas the nuclear translocation of caspases was detected already after 16 hr of incubation with cisplatin, only minor (6.6%) or no fragmentation of the nuclei was seen by DAPI staining of HeLa and Caov-4 cells, respectively. At the same time, no decrease in the levels of lamin B or full-length forms of caspases was detected. Confocal microscopy analysis also demonstrated nuclear accumulation of caspases in cells with morphologically integral nuclei.

In summary, our results suggest that, during apoptosis, initiator caspase-2, -8, -9 translocate to the nucleus in parallel with effector caspase-3, and then could participate in the nuclear degradation. Thus, further identification of the nuclear substrates of initiator caspases can shed light on the mechanisms of the demolition of the nucleus during apoptotic cell death. Given that the alterations in the nucleocytoplasmic transport system are not limited to the initiation of apoptosis, but are observed in response to various pathological and physiological stresses^3,6,66^, the nuclear entry of caspases might also occur not only during apoptotic cell death and could have far greater significance in cell metabolism. Therefore, further studies are needed to improve our understanding of initiator caspase nuclear translocation and functions.

## Materials and Methods

### Cell culture and treatment

Human cervical carcinoma HeLa cells, human ovarian carcinoma Caov-4 cells, human breast carcinoma MCF-7 cells were cultured in DMEM (Gibco) supplemented with 10% heat-inactivated FBS (Gibco) and 1% sodium pyruvate (PanEco) in the presence of 100 μg/ml penicillin and 100 μg/ml streptomycin mixture (Gibco) in a humidified 5% CO atmosphere at 37°C. To induce apoptosis, cells were treated with 35 µM of cisplatin (Teva), 10 ng/ml TNFα/5 µg/ml CHX (Sigma), or 0.1 µM staurosporine (Sigma) for the indicated time periods.

### Western blotting

Protein measurements were carried out using the Pierce BCA Protein Assay Kit (Thermo Scientific) in accordance with the manufacturer’s instructions. Samples were mixed with Laemmli’s loading buffer, boiled for 5 min, and subjected to SDS-PAGE (12%) followed by blotting onto nitrocellulose membranes for 30 min at 25 V using the Mini Trans-Blot Cell (Bio-Rad). Membranes were blocked for 1 hr with 5% non-fat milk in TBS at room temperature and subsequently probed overnight at 4°C with the primary antibody (1:1000). The following primary antibodies were used: rabbit anti-vinculin, rabbit anti-hexokinase-II, rabbit anti-Na^+^/K^+^-ATPase, rabbit anti-cleaved caspase-3 (all from Cell Signaling), mouse anti-caspase-2, mouse anti-caspase-3, mouse anti-PARP1, mouse anti-cytochrome *c* (all from BD Transduction Lab), mouse anti-lamin B, rabbit anti-Endonuclease G, mouse anti-AIF (all from Santa Cruz Biotech), mouse anti-caspase-8 (Enzo Life Science), rabbit anti-GAPDH (Trevigen), and rabbit anti-ERp29 (kindly provided by Dr. S. Mkrtchian, Karolinska Institutet). After four times washes in TBST (0.05% Tween-20 in TBS), membranes were incubated with appropriate horseradish peroxidase-conjugated secondary antibodies purchased from Cell Signaling (1:4000) for 1 hr at room temperature. Blots were developed using ECL (Amersham Biosciences) and documented using Chemi-Doc (Bio-Rad).

### Subcellular fractionation

All preparations were performed on ice. Cells were washed with PBS, harvested and resuspended in hypotonic buffer (20 mM Tris-HCl, pH 7.4, 10 mM KCl, 2 mM MgCl_2_, 1 mM EGTA, 0.5 mM DTT, 0.5 mM PMSF and Roche complete protease inhibitors; 300 μl were added per 100-mm tissue culture dish), incubated for 5 min followed by the addition of NP-40 (Nonidet P-40) to a final concentration of 0.1%. Following 3 min of incubation, the cytoplasm and nuclei were separated by centrifuging at 800 g for 8 min. Subsequently, to ensure the removal of nuclear remnants, the cytoplasmic fractions were centrifuged at 1500 g for 5 min, and the supernatants were collected as the final cytoplasmic fractions. The nuclei were purified by 10 min incubation in isotonic lysis buffer (20 mM Tris-HCl, pH 7.4, 150 mM KCl, 2 mM MgCl_2_, 1 mM EGTA, 0.3% NP-40, 0.5 mM DTT, 0.5 mM PMSF and Roche complete protease inhibitors; 500 μl were added per 100-mm tissue culture dish) and centrifuged at 700 g for 7 min. The quality of the obtained nuclei was assessed by DIC (differential interference contrast)/fluorescence microscopy using DMI6000B fluorescent microscope (Leica) following DNA staining with Hoechst33342 (1 mg/ml in PBS) (Molecular Probes) and ER (endoplasmic reticulum) staining with 1 μM ER-tracker Green (BODIPY FL Glibenclamide, Molecular Probes). For DNA digestion, the isolated nuclei were resuspended in DNAse buffer (20 mM Tris-HCl, pH 7.4, 100 mM NaCl, 4 mM MgCl_2_, 1 mM CaCl_2_, 1% NP-40, 0.5 mM DTT, 0.5 mM PMSF and Roche complete protease inhibitors) and incubated with DNAse I (Thermo Scientific) or benzonase (Sigma) for 40 min on ice.

For the determination of optimal fractionation conditions, several approaches for nuclear isolation were tested. Dounce homogenization was performed in the described above hypotonic buffer with a glass Dounce tissue grinder using 30 to 50 strokes. Subsequently, the cytoplasmic and nuclear components were separated by centrifuging at 800 g for 8 min. In case of using digitonin for cell membrane lysis, the hypotonic and isotonic solutions described above but supplemented with freshly added 0.1% digitonin were used. Sucrose purification was carried out according to the Lamond protocol (http://www.lamondlab.com/f7nucleolarprotocol.htm). Briefly, after cell membrane lysis using Dounce homogenization or NP-40 nuclear pellet was resuspended in solution S1 (0.25 M sucrose, 10 mM MgCl_2_), layered over solution S2 (0.88 M sucrose, 0.5 mM MgCl_2_) and centrifuged at 3000 g for 15 min.

### DAPI staining

Cells were grown on 13-mm round glass coverslips (Thermo Scientific). After treatment, cells were rinsed three times with PBS and fixed for 10 min in 4% paraformaldehyde. After three washes with PBS, coverslips were mounted with ProLong™ Diamond Antifade Mountant with DAPI for nuclear counterstaining (Invitrogen). The samples were examined under LSM 780 confocal laser scanner microscope (Zeiss). At least 200 cells were counted per sample to determine the percentages of cells with condensed and fragmented nuclei.

### Measurement of caspase activity

Caspase-2, -3 and -8 activities were assessed by detecting the cleavage of fluorogenic peptide substrates VDVAD-AMC, DEVD-AMC and IETD-AFC (PeptaNova), respectively. Harvested cells or nuclei isolated using the protocol described in 2.3. Subcellular fractions were resuspended with PBS supplemented with 0.5 mM PMSF and Roche complete protease inhibitors (100 μl PBS per 1 × 10^6^ cells or nuclei isolated from 2 × 10^6^ cells). 25 μl of the suspension were placed into a 96-well plate and mixed with the appropriate peptide substrate (100 μM) dissolved in 50 μl of caspase-3, -8 (100 mM HEPES, pH 7.2, 10% sucrose, 5 mM DTT, 0.001% NP-40, 0.1% CHAPS) or caspase-2 reaction buffer (100 mM MES, pH 6.5, 10% polyethylene glycol, 5 mM DTT, 0.001% NP-40, 0.1% CHAPS). Cleavage of fluorogenic peptides was monitored at 37°C using VarioScan Flash multimode detector (Thermo Scientific) by AMC or AFC liberation at 380 nm excitation and 460 nm emission, or 400 nm excitation and 505 nm emission wavelengths, respectively. The fluorescence values were normalized to protein concentrations measured using the Pierce BCA Protein Assay Kit (Thermo Scientific).

### Immunofluorescence

Cells were grown on 13-mm round glass coverslips (Thermo Scientific). After treatment, cells were rinsed three times with PBS, fixed for 10 min in 4% paraformaldehyde, washed three times with PBS and permeabilized with 0.2% Triton X-100 in PBS for 10 min. After three washing steps in PBS, blocking of nonspecific binding sites was performed by incubation of cells in 4% BSA in PBST (0.05% Triton X-100 in PBS) at 4 °С for 1 hr. Incubation with primary rabbit anti-caspase-2 (Santa Cruz Biotech, 1:150), rabbit anti-caspase-3 (Cell Signaling, 1:150), rabbit anti-caspase-8 (Thermo Scientific, 1:150) or mouse anti-caspase-9 antibodies (Thermo Scientific, 1:300) was performed overnight in 4% BSA in PBST. Then, cells were washed three times with PBST and incubated for 2 hr at 4°С with appropriate Alexa Fluor 488 secondary antibodies (Molecular Probes, 1:300) in 4% BSA in PBST. Then, after three washing steps in PBST, coverslips were mounted with ProLong™ Diamond Antifade Mountant with DAPI for nuclear counterstaining (Invitrogen). The samples were examined under LSM 780 confocal laser scanner microscope (Zeiss). ZEN software (Zeiss) was used to merge the images.

### Statistical analysis

Data are presented as means ± s.e.m. of at least 3 independent experiments. Statistical analysis was performed using Student’s t-tests at a significance level of *p < 0.005.

## Electronic supplementary material

Supplementary information

## Data Availability

All relevant data are within the paper and its Supplementary Information file.

## References

[1] Galluzzi L (2012). Molecular definitions of cell death subroutines: recommendations of the Nomenclature Committee on Cell Death 2012. Cell Death Differ..

[2] Kerr JFR, Wyllie AH, Currie AR (1972). Apoptosis: a basic biological phenomenon with wide-ranging implications in tissue kinetics. Br. J. Cancer.

[3] Ferrando-May E (2005). Nucleocytoplasmic transport in apoptosis. Cell Death Differ..

[4] Kurokawa M, Kornbluth S (2009). Caspases and Kinases in a Death Grip. Cell.

[5] Prokhorova EA, Zamaraev AV, Kopeina GS, Zhivotovsky B, Lavrik IN (2015). Role of the nucleus in apoptosis: Signaling and execution. Cell Mol Life Sci..

[6] Kopeina, G. S., Prokhorova, E. A., Zhivotovsky, B. & Lavrik, I. N. Alterations in the nucleocytoplasmic transport in apoptosis: Caspases lead the way. *Cell Prolif*., e12467 (2018). 10.1111/cpr.12467 PMC6528946 29947118

[7] Napirei M (2000). Features of systemic lupus erythematosus in Dnase1-deficient mice. Nat. Genet..

[8] Kawane K (2003). Impaired thymic development in mouse embryos deficient in apoptotic DNA degradation. Nat. Immunol..

[9] Widlak P, Garrard WT (2009). Roles of the major apoptotic nuclease-DNA fragmentation factor-in biology and disease. Cellular and Molecular Life Sciences.

[10] Nicholson DW (1995). Identification and inhibition of the ICE/CED-3 protease necessary for mammalian apoptosis. Nature.

[11] Walsh JG (2008). Executioner caspase-3 and caspase-7 are functionally distinct proteases. Proc. Natl. Acad. Sci. USA.

[12] Julien O (2016). Quantitative MS-based enzymology of caspases reveals distinct protein substrate specificities, hierarchies, and cellular roles. Proc. Natl. Acad. Sci..

[13] Benchoua A (2002). Active caspase-8 translocates into the nucleus of apoptotic cells to inactivate poly(ADP-ribose) polymerase-2. J. Biol. Chem..

[14] Besnault-Mascard L (2005). Caspase-8 sumoylation is associated with nuclear localization. Oncogene.

[15] Gu Y, Sarnecki C, Aldape RA, Livingston DJ, Su MS (1995). Cleavage of poly(ADP-ribose) polymerase by interleukin-1 beta converting enzyme and its homologs TX and Nedd 2. J. Biol. Chem..

[16] Kosugi S (2009). Six classes of nuclear localization signals specific to different binding grooves of importin alpha. J. Biol. Chem..

[17] Faleiro L, Lazebnik Y (2000). Caspases disrupt the nuclear-cytoplasmic barrier. J. Cell Biol..

[18] Kamada S, Kikkawa U, Tsujimoto Y, Hunter T (2005). Nuclear translocation of caspase-3 is dependent on its proteolytic activation and recognition of a substrate-like protein(s). J. Biol. Chem..

[19] Kamada S, Kikkawa U, Tsujimoto Y, Hunter T (2005). A-kinase-anchoring protein 95 functions as a potential carrier for the nuclear translocation of active caspase 3 through an enzyme-substrate-like association. Mol. Cell. Biol..

[20] Luo M (2010). Nuclear entry of active caspase-3 is facilitated by its p3-recognition-based specific cleavage activity. Cell Res..

[21] Colussi PA, Harvey NL, Kumar S (1998). Prodomain-dependent nuclear localization of the caspase-2 (Nedd2) precursor: A novel function for a caspase prodomain. J. Biol. Chem..

[22] Shikama Y, U M, Miyashita T, Yamada M (2001). Comprehensive studies on subcellular localizations and cell death-inducing activities of eight GFP-tagged apoptosis-related caspases. Exp. Cell Res..

[23] Baliga BC (2003). Role of prodomain in importin-mediated nuclear localization and activation of caspase-2. J. Biol. Chem..

[24] Paroni G, Henderson C, Schneider C, Brancolini C (2002). Caspase-2 can trigger cytochrome C release and apoptosis from the nucleus. J. Biol. Chem..

[25] Ritter PM (2000). Nuclear localization of procaspase-9 and processing by a caspase-3-like activity in mammary epithelial cells. Eur. J. Cell Biol..

[26] Janssens S, Tinel A (2012). The PIDDosome, DNA-damage-induced apoptosis and beyond. Cell Death Differ..

[27] Ando K (2017). NPM1 directs PIDDosome-dependent caspase-2 activation in the nucleolus. J Cell Biol..

[28] Tinnikov AA, Samuels HH (2013). A novel cell lysis approach reveals that caspase-2 rapidly translocates from the nucleus to the cytoplasm in response to apoptotic stimuli. Plos One.

[29] Lopez-Cruzan M (2016). Caspase-2 resides in the mitochondria and mediates apoptosis directly from the mitochondrial compartment. Cell Death Discov..

[30] Krumschnabel G, Sohm B, Bock F, Manzl C, Villunger A (2009). The enigma of caspase-2: the laymen’s view. Cell Death Differ..

[31] Mancini M (2000). Caspase-2 is localized at the Golgi complex and cleaves Golgin-160 during apoptosis. J. Cell Biol..

[32] van Loo G (2002). Caspases are not localized in mitochondria during life or death. Cell Death Differ..

[33] Bouchier-Hayes L, Sidi S (2017). The nucleolus: A new home for the PIDDosome. Cell Cycle.

[34] Seibel NM, Eljouni J, Nalaskowski MM, Hampe W (2007). Nuclear localization of enhanced green fluorescent protein homomultimers. Anal. Biochem..

[35] Faragher AJ (2007). Death receptor-induced apoptosis reveals a novel interplay between the chromosomal passenger complex and CENP-C during interphase. Mol. Biol. Cell.

[36] Inoue S, Browne G, Melino G, Cohen GM (2009). Ordering of caspases in cells undergoing apoptosis by the intrinsic pathway. Cell Death Differ..

[37] Hetzer MW (2010). The nuclear envelope. Cold Spring Harb. Perspect. Biol..

[38] Dasari S, Bernard Tchounwou P (2014). Cisplatin in cancer therapy: Molecular mechanisms of action. Eur J Pharmacol..

[39] Tristan C, Shahani N, Sedlak TW, Sawa A (2011). The diverse functions of GAPDH: Views from different subcellular compartments. Cell. Signal..

[40] Ahuatzi D, Herrero P, De La Cera T, Moreno F (2004). The Glucose-regulated Nuclear Localization of Hexokinase 2 in Saccharomyces cerevisiae Is Mig1-dependent. J. Biol. Chem..

[41] Ahuatzi D, Riera A, Peláez R, Herrero P, Moreno F (2007). Hxk2 regulates the phosphorylation state of Mig1 and therefore its nucleocytoplasmic distribution. J. Biol. Chem..

[42] Sirover MA (2012). Subcellular dynamics of multifunctional protein regulation: Mechanisms of GAPDH intracellular translocation. J. Cell. Biochem..

[43] Saelens X (2004). Toxic proteins released from mitochondria in cell death. Oncogene.

[44] Wang Y (2016). A nuclease that mediates cell death induced by DNA damage and poly(ADP-ribose) polymerase-1. Science.

[45] Soldani C, Bottone MG, Pellicciari C, Scovassi AI (2001). Two-color fluorescence detection of poly (ADP-Ribose) polymerase-1 (PARP-1) cleavage and DNA strand breaks in etoposide-induced apoptotic cells. Eur. J. Histochem..

[46] Soldani C (2001). Poly(ADP-ribose) polymerase cleavage during apoptosis: When and where?. Exp. Cell Res..

[47] Perdoni F (2009). Distribution of centromeric proteins and PARP-1 during mitosis and apoptosis. Ann N Y Acad Sci..

[48] Errami Y (2013). Apoptotic DNA fragmentation may be a cooperative activity between caspase-activated deoxyribonuclease and the poly(ADP-ribose) polymerase-regulated DNAS1L3, an endoplasmic reticulum-localized endonuclease that translocates to the nucleus during apoptosis. J. Biol. Chem..

[49] Laukoter S (2017). Differences in T cell cytotoxicity and cell death mechanisms between progressive multifocal leukoencephalopathy, herpes simplex virus encephalitis and cytomegalovirus encephalitis. Acta Neuropathol..

[50] Chen Y (2018). Bcl-2 protects TK6 cells against hydroquinone-induced apoptosis through PARP-1 cytoplasm translocation and stabilizing mitochondrial membrane potential. Environ. Mol. Mutagen..

[51] Roser S, Pool-Zobel BL, Rechkemmer G (2001). Contribution of apoptosis to responses in the comet assay. Mutat. Res. - Genet. Toxicol. Environ. Mutagen..

[52] Joseph B (2001). Defective caspase-3 relocalization in non-small cell lung carcinoma. Oncogene.

[53] Damarla M (2014). Mitogen-activated protein kinase-activated protein kinase 2 mediates apoptosis during lung vascular permeability by regulating movement of cleaved caspase 3. Am. J. Respir. Cell Mol. Biol..

[54] Cao G (2001). Caspase-activated DNase/DNA fragmentation factor 40 mediates apoptotic DNA fragmentation in transient cerebral ischemia and in neuronal cultures. J. Neurosci..

[55] Jänicke RU, Sprengart ML, Wati MR, Porter AG (1998). Caspase-3 Is Required for DNA Fragmentation and Associated with Apoptosis. J. Biol. Chem..

[56] Zhivotovsky B, Samali A, Gahm A, Orrenius S (1999). Caspases: their intracellular localization and translocation during apoptosis. Cell Death Differ..

[57] Boege Y (2017). Dual Role of Caspase-8 in Triggering and Sensing Proliferation-Associated DNA Damage, a Key Determinant of Liver Cancer Development. Cancer Cell.

[58] Baliga BC, Read SH, Kumar S (2004). The biochemical mechanism of caspase-2 activation. Cell Death Differ..

[59] Riedl SJ (2001). Structural basis for the activation of human procaspase-7. Proc. Natl. Acad. Sci. USA.

[60] Zamaraev AV, Kopeina GS, Prokhorova EA, Zhivotovsky B, Lavrik IN (2017). Post-translational Modification of Caspases: The Other Side of Apoptosis Regulation. Trends Cell Biol..

[61] Thompson CB (1995). Apoptosis in the pathogenesis and treatment of disease. Science.

[62] Agard NJ (2012). Global kinetic analysis of proteolysis via quantitative targeted proteomics. Proc. Natl. Acad. Sci..

[63] Yao Z, Duan S, Hou D, Heese K, Wu M (2007). Death effector domain DEDa, a self-cleaved product of caspase-8/Mch5, translocates to the nucleus by binding to ERK1/2 and upregulates procaspase-8 expression via a p53-dependent mechanism. EMBO J..

[64] Slee EA, Adrain C, Martin SJ (2001). Executioner Caspase-3, -6, and -7 Perform Distinct, Non-redundant Roles during the Demolition Phase of Apoptosis. J. Biol. Chem..

[65] Arutyunova E (2013). Localization of non-native D-glyceraldehyde-3-phosphate dehydrogenase in growing and apoptotic HeLa cells. Biochemistry (Mosc).

[66] Kodiha M, Stochaj U (2012). Nuclear Transport: A Switch for the Oxidative Stress—Signaling Circuit?. J. Signal Transduct..

