# Supplementary material for: Apoptosis regulation by subcellular relocation of caspases
Source: Sci Rep. 2018 Aug 15;8:12199. doi: 10.1038/s41598-018-30652-x (PMC6093910; doi:10.1038/s41598-018-30652-x)
Supplement: Supplementary file 1 — Supplementary information [file 41598_2018_30652_MOESM1_ESM.pdf]

Supplementary Information

Apoptosis regulation by subcellular relocation of caspases

Evgeniia A. Prokhorova[1,2,#], Gelina S. Kopeina[1,#], Inna N. Lavrik[1,3], and Boris Zhivotovsky[1,4,*]

[1]Faculty of Fundamental Medicine, Lomonosov Moscow State University, 119991 Moscow, Russia;

[2]Present address: Sir William Dunn School of Pathology, University of Oxford, OX1 3RE Oxford, UK;

[3]Translational Inflammation Research, Otto von Guericke University, 39106 Magdeburg, Germany;

[4]Division of Toxicology, Institute of Environmental Medicine, Karolinska Institutet, Box 210, 17177 Stockholm, Sweden

#These authors equally contributed to this work

[*]Corresponding author: Professor Boris Zhivotovsky, Institute of Environmental Medicine, Karolinska Institutet, Box 210, 17177 Stockholm, Sweden
E-mail: Boris.Zhivotovsky@ki.se

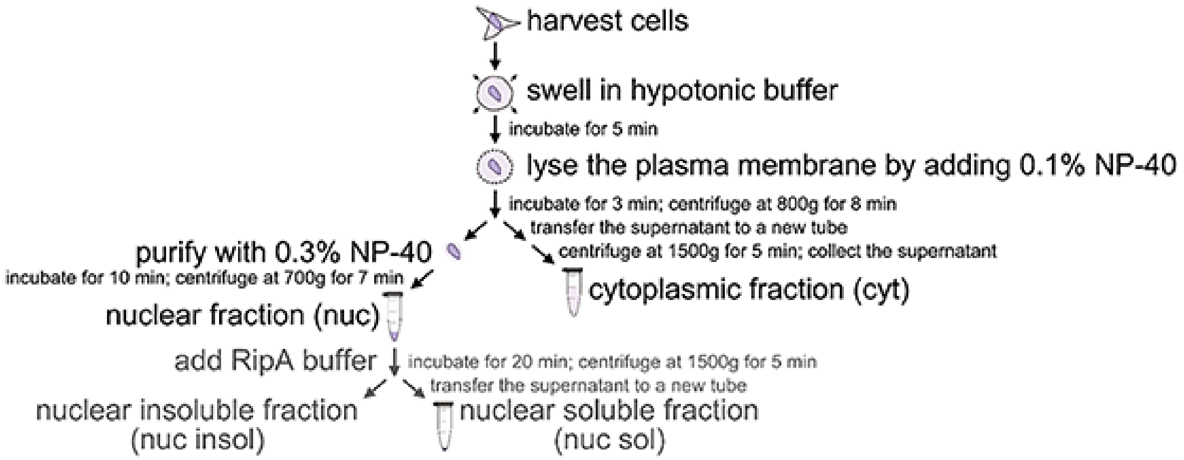

Figure S1 Schematic representation of the fractionation protocol.

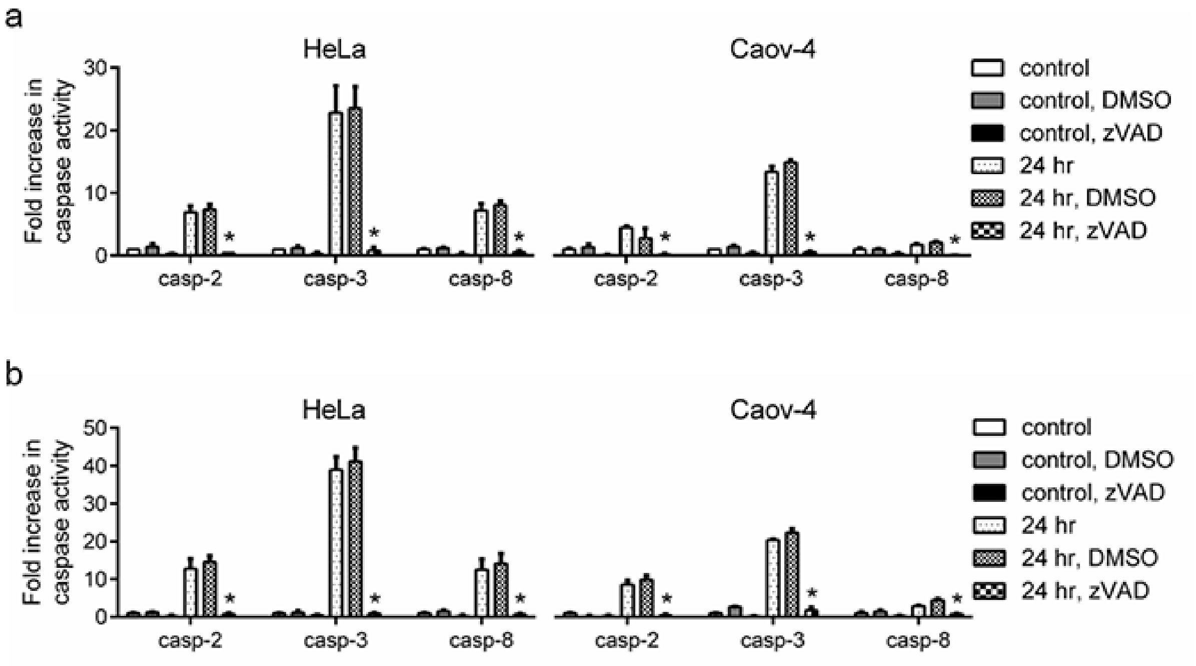

Figure S2 Reduction in caspase activity in the presence of pan-caspase inhibitor z-VAD-fmk. Enzyme activity was monitored in the isolated nuclei **(a)** and cells **(b)** at the indicated time points by the release of the fluorogenic group AMC from VDVAD-AMC, fold increase in caspase-2 activity, and DEVD-AMC, fold increase in caspase-3-like activity; by the release of AFC from IETD-AFC, fold increase in caspase-8 activity. Pretreatment with z-VAD-fmk at the concentration of 20 μM for 1 hr prior to exposure to cisplatin (35 μM, 24 hr) was conducted. Results represent the means±s.e.m. of at least 3 independent experiments. * $p<0.005$.

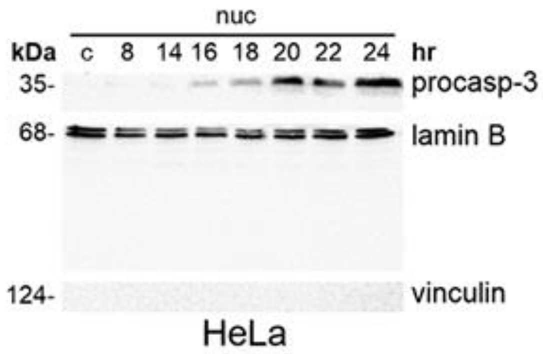

Figure S3 Western blot analysis of lamin B in the nuclear lysates of HeLa cells treated with cisplatin (35 μM).

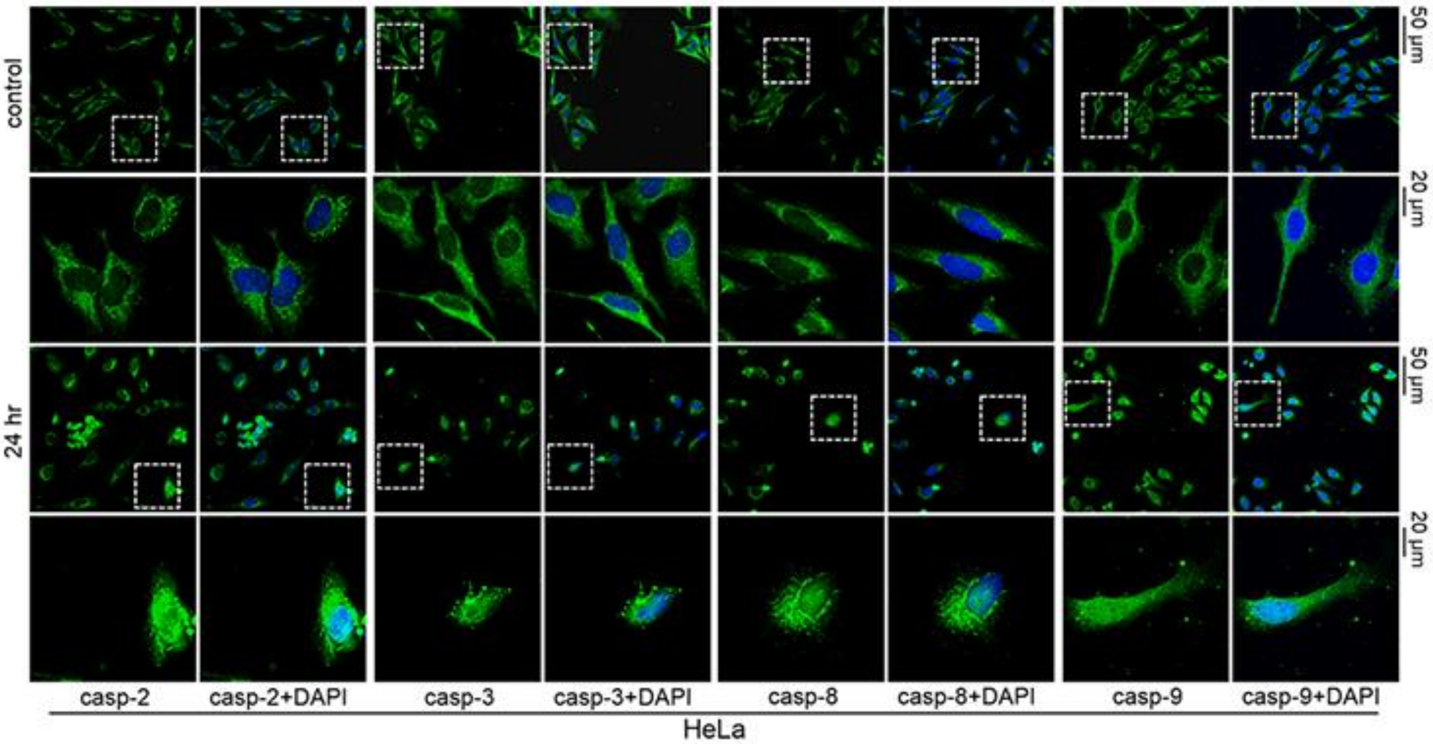

Figure S4 Representative images of HeLa cells stained with primary anti-caspase-2, -3, -8 or -9 and secondary Alexa Fluor 488 (green) antibodies obtained for the analysis of caspase redistribution following cisplatin treatment (35 μM, 24 hr). Nuclei were counterstained with DAPI (blue). Scale bars, 50 and 20 μm.

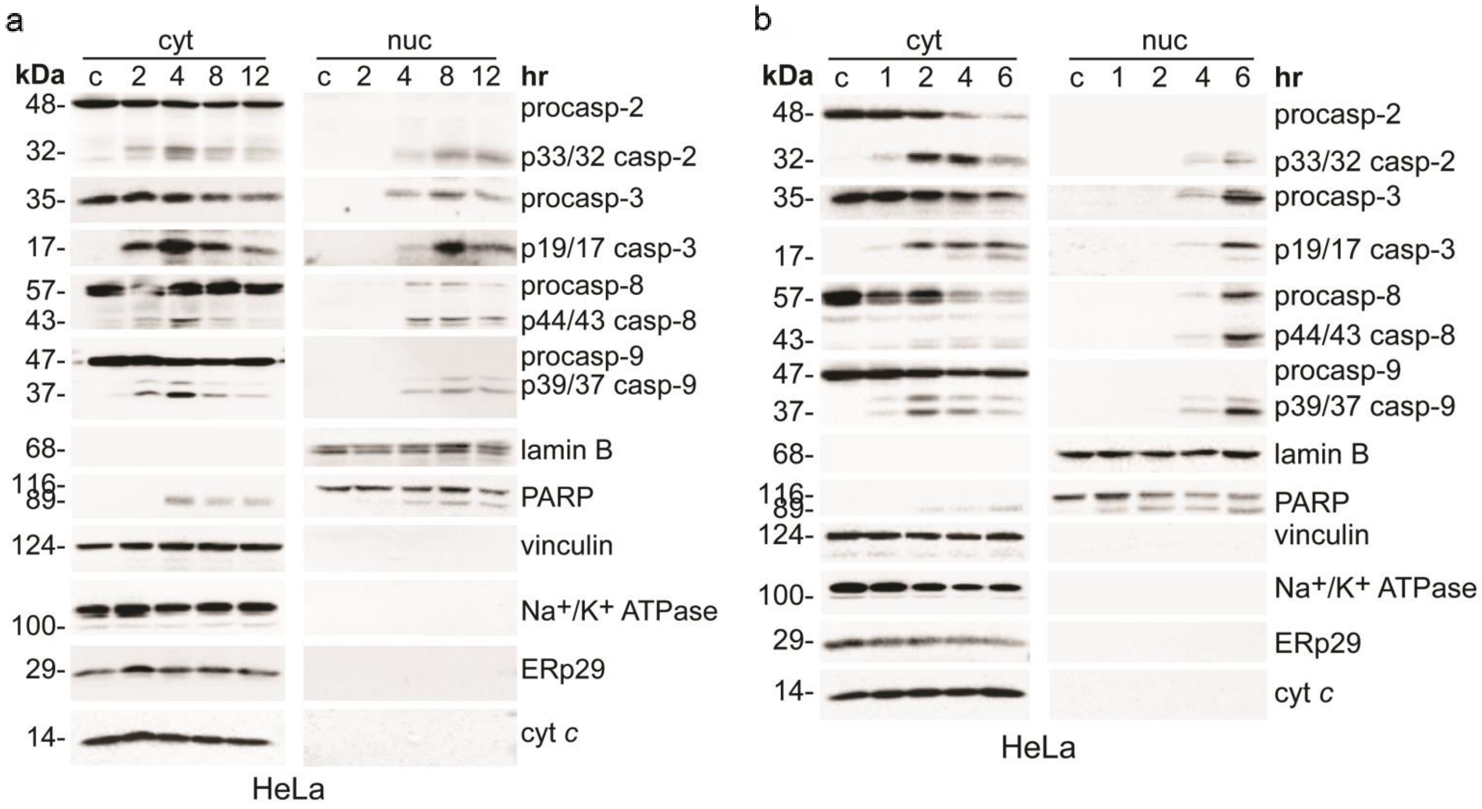

Figure S5 Functionally active initiator caspase-2, -8, -9 and executioner caspase-3 accumulate in the nucleus in response to the treatment of HeLa cells with TNFα (10 ng/ml)/CHX (5 μg/ml) **(a)** or staurosporine (0.1 μM) **(b)**. Representative images of three independent experiments are shown.

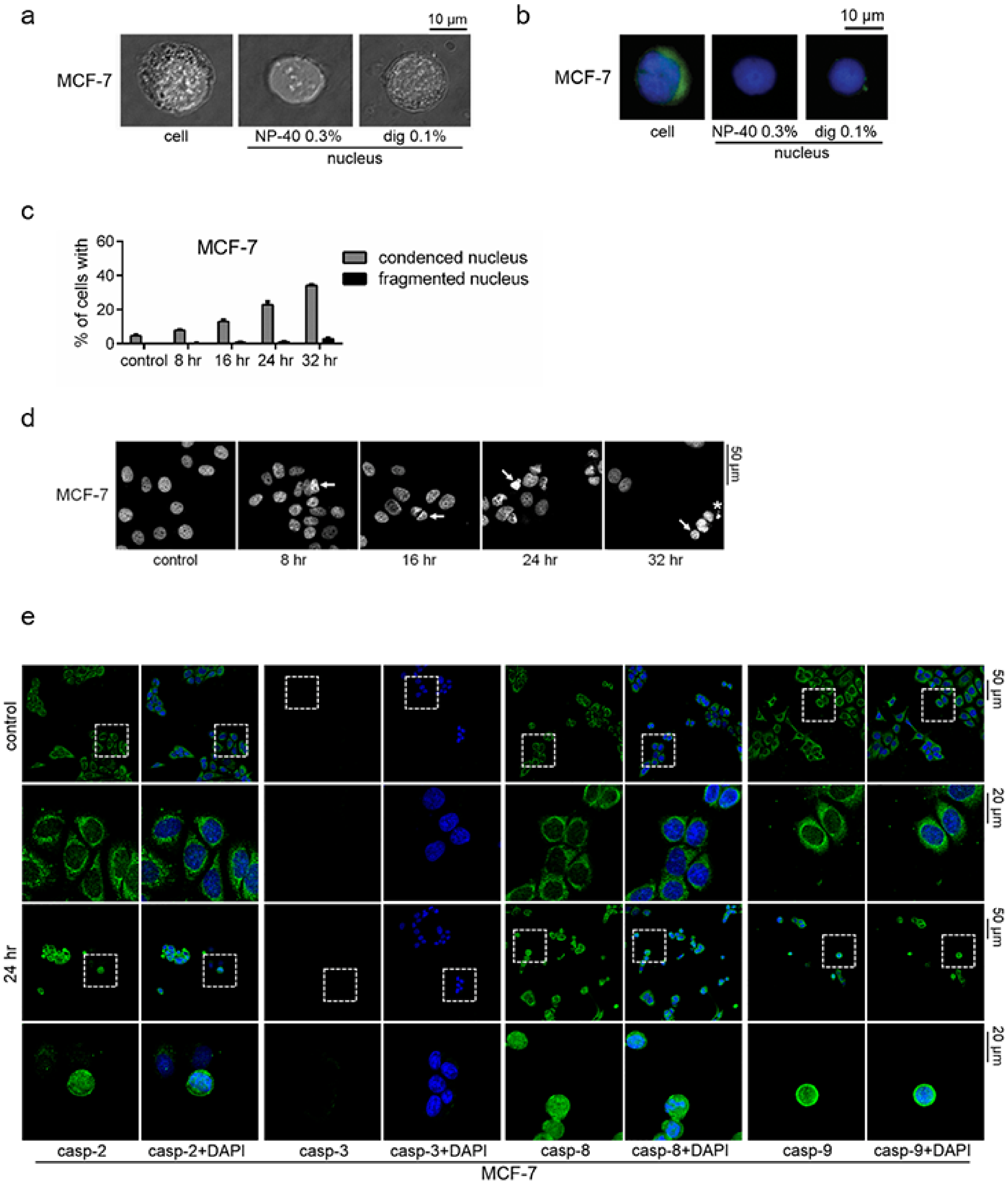

Figure S6 (a) MCF-7 cells and isolated nuclei stained with Hoechst33342 and imaged by dual DIC and fluorescence microscopy. **(b)** MCF-7 cells and isolated nuclei stained with Hoechst33342 and ER-tracker Green (BODIPY FL Glibenclamide) and imaged by

fluorescence. **(c, d)** Cisplatin (35 μM)-induced changes in the morphology of MCF-7 cells. **(c)** Percentages of cells with condensed and fragmented nuclei detected by DAPI staining. Results are shown as mean±s.e.m. of 3 independent experiments. At least 200 stained cells were counted in each experiment. **(d)** Representative images obtained using confocal microscopy. The white arrows and asterisk mark examples of condensed and fragmented nuclei, respectively. Scale bar, 50 μm. **(e)** Representative images of MCF-7 cells stained with primary anti-caspase-2, -8 or -9 and secondary Alexa Fluor 488 (green) antibodies obtained for the analysis of caspase redistribution following cisplatin treatment (35 μM, 24 hr). Nuclei were counterstained with DAPI (blue). Scale bars, 50 and 20 μm.
